# Composition of Different Amalgams

**Published:** 1883-10

**Authors:** 


### COMPOSITION OF DIFFERENT AMALGAMS.

Arrington amalgam, silver 40 per cent, tin 60 per cent.

Diamond amalgam, silver 31.76, tin 66.74, gold 1.50.

Hood's amalgam, silver 34.64, tin 60.37, gold 2.70, iron 2.90.

Johnsohn & Lund's amalgam, silver 38.27, tin 59.58, platinum 1.34, gold 0.81.

Lawrence's amalgam, silver 47.87, tin 33.68, copper 14.91, gold 3.54.

Moffitt's amalgam, silver 35.17, tin 62.01, gold 2.82.

Townsend's amalgam, silver 40.21, tin 47.54, copper 10.65, gold 1.6.

Townsend's improved amalgam, silver 39.00, tin 55.65, gold 5.31.

Walker's amalgam, silver 34.89, tin 60.01, platinum 0.96, gold 4.14.—*Monatsschrift des Vereins deutscher Zahnkuenstler.*

